# Erysipelas

**Published:** 1890-11

**Authors:** G. M. Pease

**Affiliations:** San Francisco, Cal.


### ERYSIPELAS.

G. M. Pease, M. D., San Francisco, Cal.

(Report of Bureau of Clinical Medicine, I. H. A.)

Recently there has fallen to my lot a case which presented some unusual conditions, symptoms of directly opposite character upon opposite sides of the body, but which yielded promptly after the selection of the proper remedy.

Mrs. M——, aged about sixty-five. For many years has been subject to attacks of erysipelas, nearly always in the same localities, but previously she had been troubled with tonsillitis, for both of which she received good old-fashioned "regular" treatment. From three to four weeks she was "laid up" and for weeks afterward was not strong.

About four years ago I attended her during one of these attacks of erysipelas, but did not consider that I had done any very brilliant work in the case, though she seemed much pleased with the result, because, though lasting three weeks, she had not suffered as much, had not been heavily dosed nor subjected to the inconvenience of external applications, and when the local symptoms were gone she did not feel as much prostrated as formerly.

And now there has been an interim of four years against the former almost yearly attacks.

It is possible that this last attack had an outcropping a week before I saw her, as her husband came to me with a description of her sore throat. He did not think it necessary for me to see her, and as he gave a good picture of Merc. I sent that remedy in the thirtieth potency. The effect was good, for in twenty-four hours she felt all right.

May 25th. I was called to see her, the following symptoms presenting. There was a red spot covering the point of the left elbow, extending half way around the arm and upward and downward about two inches. Upon the outer part of the left forearm another red spot about the size of a half dollar, upon the wrist another, on the leg and ankle other small spots. These were of a phlegmonous character, very hot and extremely tender to the touch, but not especially painful on motion. Upon the right side at the wrist a small spot, very red, with a line of red extending up the arm about two inches; one of the fingers at the metacarpal joint was slightly red and swollen. The right knee was alike affected. These points were not as hot nor were they phlegmonous, but were more sensitive than upon the other side, and the least motion caused great pain. This side she kept uncovered because heat aggravated it, but the left side was carefully wrapped in flannel because heat made it better. Motion aggravated all the pains—she was very thirsty, wanted large quantities of water. Thinking Bryonia was best indicated, I gave it in the 200th potency. The following day there was a slight amelioration of the right side symptoms, but the left side was decidedly worse, phlegmonous spots larger and more of them. No change of remedy.

Third day.—Right side better so far as the rheumatic character of the pains was concerned, but phlegmonous spots had appeared here also and nearly the whole of the left arm was covered. There was no vesicular appearance at any point. The right side was still uncovered, while the left demanded all the heat it could get.

She felt somewhat restless but could not move because it increased pain. I *may* not have previously looked at the tongue but to-day I did, and found it heavily coated except a triangle at the tip, the base being at the end of the tongue and the point inward; this triangle was very dark red, quite dry, while the rest of the tongue did not look dry. This condition of tongue I have always associated with Rhus-tox. and Sulphur. Taking into consideration the other symptoms it was an easy matter to decide between the two, and Rhus-tox.$^{200}$ was given.

Fourth day.—As I entered the room my patient greeted me with a smile, and held out her left hand as if to shake hands, but withdrew it and extended the right.

Words were not needed to define her actions, but she used them freely to expresss her feelings of satisfaction.

The phlegmonous condition had very greatly disappeared, the surface less red; scarcely any heat; did not need one side covered more than the other; had slept well all night, and now asked what she could have to eat. Remedy, Sac-lac.

Fifth day.—As far as my professional services were concerned there was no necessity for my visit, scarcely a vestige of the late trouble being present.

Comments.—Possibly the earlier inspection of the tongue might have saved the patient a couple of days' suffering, because it was the red tip which caused a change in prescription, but I am not prepared to say positively that I did not see it at an earlier stage because it is such a natural thing to ask to see the tongue.

According to Allen's *Symptom Register* there are a number of remedies having redness at the tip of the tongue, prominently noted being Argent-n., Ars., Phyt., and Rhus-tox., but he does not include Sulph.

Many observations have fixed upon my memory the well-defined *triangle* as belonging to Rhus and Sulph., because other symptoms had so clearly indicated one or the other when that triangle was present, the most clearly marked triangle demanding Rhus. This decided contradiction between the two sides was peculiar. Was the first remedy a mistake? It was chosen to combat what appeared to be the most distressing symptoms; that it had some effect was evident.

Bry. and Rhus being complementary to so great an extent, it often happens in my experience that when one has been given the other may be needed to complete the cure.

